# New surgical scoring system to predict postoperative mortality

**DOI:** 10.1007/s00540-016-2290-2

**Published:** 2016-12-19

**Authors:** Maho Kinoshita, Nobutada Morioka, Mariko Yabuuchi, Makoto Ozaki

**Affiliations:** Department of Anesthesiology, Tokyo Women’s Medical University, Shinjuku, Tokyo, 1628666 Japan

**Keywords:** Surgical Apgar score (sAs), American Society of Anesthesiologists physical status classification (ASA-PS), Postoperative mortality, Patient safety

## Abstract

**Purpose:**

There is still no easy and highly useful method to comprehensively assess both preoperative and intraoperative patient statuses to predict postoperative outcomes. We attempted to develop a new scoring system that would enable a comprehensive assessment of preoperative and intraoperative patient statuses instantly at the end of anesthesia, predicting postoperative mortality.

**Methods:**

The study included 32,555 patients who underwent surgery under general or regional anesthesia from 2008 to 2012. From the anesthesia records, extracted factors, including patient characteristics and American Society of Anesthesiologists physical status classification (ASA-PS), and three intraoperative indexes (the lowest heart rate, lowest mean arterial pressure, and estimated volume of blood loss) are used to calculate the surgical Apgar score (sAs). The sAs and ASA-PS, and surgical Apgar score combined with American Society of Anesthesiologists physical status classification (SASA), which combines the sAs and ASA-PS into a single adjusted scale, were compared and analyzed with postoperative 30-day mortality.

**Results:**

Increased severity of the sAs, ASA-PS and SASA was correlated with significantly higher mortality. The risk of death was elevated by 3.65 for every 2-point decrease in the sAs, by 6.4 for every 1-point increase in the ASA-PS, and by 9.56 for every 4-point decrease in the SASA. The ROC curves of the sAs and ASA-PS alone also individually demonstrated high validity (AUC = 0.81 for sAs and 0.79 for ASA-PS, *P* < 0.001). The SASA was even more valid (AUC = 0.87, *P* < 0.001).

**Conclusions:**

The sAs and ASA-PS were shown to be extremely useful for predicting 30-day mortality after surgery. An even higher predictive ability was demonstrated by the SASA, which combines these simple and effective scoring systems.

## Introduction

In recent years, with advances in the fields associated with anesthesia, surgery has become increasingly applicable to a wider range of diseases and patients, and the annual number of operations performed is also increasing globally [1]. In terms of patient safety and medical economics, an important issue is how to reduce the incidence of perioperative complications and mortality. At least half of postoperative complications can be prevented, while improvements in anesthesia-associated factors contribute greatly to the prevention of complications [2–4]. Thus, many assessment methods to estimate the incidence of postoperative complications and postoperative mortality have been proposed. Among them, the acute physiology and chronic health evaluation (APACHE), the physiological and operative severity score for the enumeration of mortality and morbidity (POSSUM), and others have been reported to be highly useful, and many revised versions with improved accuracy have been reported [5–7]. However, while these methods have been designed for presumptive use in the field of intensive care, the large number of essential test items and complex calculation procedures have been problematic. Thus, these methods are unsuitable for immediate calculation of scores after surgery, identification of patients at high risk, and determination of intensive care unit (ICU) admissions. These methods have not been widely adopted for predicting postoperative outcomes. Conventionally, the American Society of Anesthesiologists physical status classification (ASA-PS) is well known for its simplicity. However, it is problematic that this classification system depends largely on the subjective judgment of evaluators and is also broadly divided into categories [8]. In addition, ASA-PS scores are determined without consideration of surgical invasiveness and other intraoperative factors, but only based on preoperative patient status. For these reasons, although the ASA-PS is simple and useful for assessing preoperative physical status, this scoring system has been regarded as insufficient for predicting postoperative outcomes. Against this background, Gawande et al. proposed the surgical Apgar score (sAs) (Table 1), which was named after the obstetric Apgar score, in 2007 [9]. This new scoring system, in which scores are calculated from only 3 intraoperative factors (lowest intraoperative heart rate, lowest mean intraoperative blood pressure, and volume of intraoperative blood loss), attracted attention for its simplicity. Subsequently, this scoring system has been shown to be highly useful for predicting the incidence of postoperative complications and postoperative mortality in many surgical specialties beyond general and vascular surgery, for which the system was originally developed [10]. However, in contrast to the ASA-PS, the sAs is calculated as a score mainly based on intraoperative patient status, and does not directly incorporate an assessment of preoperative patient status. There is still no easy and highly useful method for comprehensively assessing both preoperative and intraoperative patient statuses to predict postoperative outcomes.

**Table 1:** Surgical Apgar score used in this study

	Surgical Apgar score, no. of points				
	0	1	2	3	4
Estimated blood loss (ml)	>1000	601–1000	101–600	≤100	
Lowest mean arterial pressure (mmHg)	<40	40–54	55–69	≥70	
Lowest heart rate (min)	>85	76–85	66–75	56–65	≤55

This table was prepared from the article written by Gawande et al. [9]

In this study, we attempted to develop a new scoring system that would enable a comprehensive assessment of preoperative and intraoperative patient statuses instantly following entry of data into an electronic anesthesia chart, accurately and automatically predicting postoperative mortality. The usefulness of our new scoring system was compared and analyzed with that of the sAs and ASA-PS, which are the components of our new system.

## Materials and methods

### Subjects

Ethical approval for this study (Approval number 2521) was provided by the Ethical Committee of Tokyo Women’s Medical University, Tokyo, JAPAN (Chairman Prof S. Miyazaki) on 25 June 2012. In addition, this study was registered under the University Hospital Medical Information Network- Clinical Trial Registry (UMIN-CTR) (unique trial number: UMIN000016990). The study included 32,555 patients who underwent surgery under general or regional anesthesia at Tokyo Women’s Medical University Hospital between February 1, 2008, and February 29, 2012.

### Exclusion criteria

The following patients were excluded: those aged 16 years or younger, those undergoing cardiovascular surgery, those receiving electroconvulsive therapy, those undergoing magnetic resonance imaging-guided brain surgery, those receiving anesthesia management outside of an operating room, and those in whom no anesthesiologist was involved in anesthesia management.

### Protocol

In all patients, factors presumably associated with surgical outcomes, including patient characteristics and ASA-PS scores, were extracted from the Anesthesia Information Management Systems (AIMS) (MetaVision: FUKUDA DENSHI, Tokyo, Japan). In addition, the lowest heart rate, lowest mean arterial pressure, and estimated volume of blood loss were extracted to calculate the sAs (Table 1). Among the extracted intraoperative biological data, all data showing a lowest heart rate of 40 bpm or lower and a lowest mean arterial pressure of 40 mmHg or lower were individually confirmed with each anesthesia chart to determine whether they were outliers due to artifacts or not. These data were manually corrected and entered. Whether or not each patient had died within 30 days of surgery was determined from the patients’ medical records. The sAs and ASA-PS scores were calculated from the data extracted from the AIMS. We develped a new scoring system (the SASA) by combining the surgical Apgar score (sAs) with American Society of Anesthesiologists physical status classification (ASA-PS), using the follow equation.

$${\text{SASA}} = {\text{sAs}} + \left( {6 - {\text{ASA-}}  {\text{PS}}} \right){ \times }2.$$

As the ASA-PS score increases, severity increases. Conversely, as the sAs score decreases, severity increases. While the ASA-PS is rated on a 5-point scale except for a patient declared brain dead considered to be ASA-PS VI, the sAs is on a 10-point scale. In the equation above, the ASA-PS score is subtracted from 6 to make its score mean the same tendency of severity as sAs and multiplied by 2 to equalize the scale of points between the sAs and ASA-PS scores, which are then combined. We thought that ASA-PS VI should be excluded from this equation because their mortality is 100%. As a primary endpoint of this study the sAs, ASA-PS, and SASA were compared and analyzed to determine whether the scores were associated with postoperative 30-day mortality. The association of other factors, including patient characteristics, with postoperative 30-day mortality was analyzed as a secondary endpoint of the study.

The original identification (ID) numbers assigned to the AIMS data and patient medical records used for each analysis were converted according to certain rules so that the modified ID numbers could not lead to identification of individual patients or provide access to the original data.

### Statistical analysis

Data are presented as means and standard deviations, medians with interquartile range, or frequencies. One-way analysis of variance (ANOVA) was used to compare normally distributed continuous variables among groups and the Kruskal–Wallis *H* test was used for skewed continuous or ordinal discrete variables. The chi-square test was used to compare nominal variables. To evaluate the impact of sAs and ASA-PS on 30-day mortality, univariate and multivariate logistic regression models were used. The interaction and multicollinearity in the model were assessed using regression diagnostic analysis. To compare the diagnostic performance of the three scores, SASA, sAs, and ASA-PS, receiver operating characteristic (ROC) curves were used. Two-tailed *P* values of less than 0.05 were considered statistically significant. Analyses were performed with the SAS system ver. 9.3 (SAS Institute, Cary, NC) at an independent biostatistics and data center (STATZ Institute, Inc., Tokyo, Japan).

## Results

Of the total 32,555 patients, 2808 with incomplete data and 5429 meeting the exclusion criteria were excluded. The remaining 24,318 patients were analyzed. Patients underwent surgery in the following specialities: gastroenterological surgery, urology, obstetrics and gynecology, neurosurgery, general surgery, plastic reconstructive surgery, orthopedic surgery, endocrine surgery, thoracic surgery, otorhinolaryngology, oral surgery, emergency and critical care center, and other.

### Characteristics of the sAs, ASA-PS, and SASA

None of the 3 scoring systems were associated with age, sex, body mass index (BMI), surgical specialties, operative duration, or anesthesia duration. The rate of emergency surgery increased as severity increased, with lower sAs, with higher ASA-PS and with lower SASA: however, it was not significant (Tables 2, 3, 4).

**Table 2:** The Surgical Apgar score (sAs) according to perioperative factors and 30-day mortality

	Total (*N* = 24,318)	0–2 (*N* = 156)	3–4 (*N* = 1086)	5–6 (*N* = 3903)	7–8 (*N* = 11,906)	9–10 (*N* = 7267)	*P* value
ASA-PS							
1	6293 (25.9%)	13 (8.3%)	172 (15.8%)	634 (16.2%)	3049 (25.6%)	2425 (33.4%)	<0.001
2	13,782 (56.7%)	54 (34.6%)	581 (53.5%)	2260 (57.9%)	6851 (57.5%)	4036 (55.5%)	
3	4113 (16.9%)	70 (44.9%)	307 (28.3%)	979 (25.1%)	1965 (16.5%)	792 (10.9%)	
4	123 (0.5%)	17 (10.9%)	25 (2.3%)	28 (0.7%)	40 (0.3%)	13 (0.2%)	
5	7 (0.0%)	2 (1.3%)	1 (0.1%)	2 (0.1%)	1(0.0%)	1 (0.0%)	
Age (years)	55.2 ± 17.1	50.8 ± 17.0	53.6 ± 1 7.8	56.8 ± 17.0	55.9 ± 16.9	53.4 ± 17.3	<0.001
Women	13,020 (53.5%)	68 (43.6%)	607 (55.9%)	1972 (50.5%)	6452(54.2%)	3921 (54.0%)	<0.001
BMI (kg/m^2^)	22.4 ± 3.8	22.7 ± 3.7	23.0 ± 4.1	22.5 ± 4.0	22.4 ± 3.7	22.4 ± 3.6	<0.001
Estimated blood loss							
Median [IQR] (ml)	40 [10–190]	1569 [1068–2923]	1077 [581–1608]	350 [100–781]	40 [10–140]	15 [5–40]	<0.001
>1000	1337 (5.5%)	127 (81.4%)	597 (55.0%)	602 (15.4%)	11 (0.1%)	–	
601–1000	1317 (5.4%)	20 (12.8%)	214 (19.7%)	830 (21.3%)	253 (2.1%)	–	<0.001
101–600	5324 (21.9%)	9 (5.8%)	183 (16.9%)	1486 (38.1%)	3377 (28.4%)	269 (3.7%)	
≤100	16,340 (67.2%)	–	92 (8.5%)	985 (25.2%)	8265 (69.4%)	6998 (96.3%)	
Lowest mean arterial pressure							
Mean ± SD (mmHg)	56.9 ± 12.9	43.3 ± 11.4	49.0 ± 12.3	50.7 ± 12.2	53.9 ± 10.7	66.7 ± 10.8	<0.001
<40	1247 (5.1%)	61 (39.1%)	248 (22.8%)	552 (14.1%)	386 (3.2%)	–	
40–54	9956 (40.9%)	77 (49.4%)	556 (51.2%)	2293 (58.7%)	7030 (59.0%)	–	<0.001
55–69	9767 (40.2%)	18 (11.5%)	229 (21.1%)	817 (20.9%)	3789 (31.8%)	4914 (67.6%)	
≥70	3348 (13.8%)	–	53 (4.9%)	241 (6.2%)	701 (5.9%)	2353 (32.4%)	
Lowest heart rate							
Mean ± SD (bpm)	54.6 ± 10.8	87.1 ± 13.5	72.2 ± 14.7	62.3 ± 11.9	53.4 ± 8.3	49.1 ± 5.8	<0.001
>85	426 (1.8%)	80 (51.3%)	218 (20.1%)	128 (3.3%)	–	–	
76–85	707 (2.9%)	52 (33.3%)	205 (18.9%)	353 (9.0%)	97 (0.8%)	–	
66–75	2189 (9.0%)	24 (15.4%)	304 (28.0%)	1075 (27.5%)	786 (6.6%)	–	<0.001
56–65	5951 (24.5%)	–	276 (25.4%)	1291 (33.1%)	3738 (31.4%)	646 (8.9%)	
≤55	15,045 (61.9%)	–	83 (7.6%)	1056 (27.1%)	7285 (61.2%)	6621 (91.1%)	
Emergency procedure	2525 (10.4%)	113 (72.4%)	396 (36.5%)	753 (19.3%)	931 (7.8%)	332 (4.6%)	<0.001
Operative duration (h)	2.7 ± 1.9	3.1 ± 2.4	3.6 ± 2.8	3.5 ± 2.3	2.8 ± 1.8	2.1 ± 1.4	<0.001
Anesthesia duration (h)	3.7 ± 2.2	3.9 ± 3.0	4.5 ± 3.4	4.5 ± 2.7	3.9 ± 2.0	3.1 ± 1.6	<0.001
30-Day mortality	123 (0.51%)	18 (11.54%)	32 (2.95%)	37 (0.95%)	29 (0.24%)	7 (0.10%)	<0.001

**Table 3:** The American Society of Anesthesiologists physical status (ASA-PS) according to perioperative factors and 30-day mortality

	Total (*N* = 24,318)	1 (*N* = 6293)	2 (*N* = 13,782)	3 (*N* = 4113)	4 (*N* = 123)	5 (*N* = 7)	*P* value
sAs							
0–2	156 (0.6%)	13 (0.2%)	54 (0.4%)	70 (1.7%)	17 (13.8%)	2 (28.6%)	<0.001
3–4	1086 (4.5%)	172 (2.7%)	581 (4.2%)	307 (7.5%)	25 (20.3%)	1 (14.3%)	
5–6	3903 (16.0%)	634 (10.1%)	2260 (16.4%)	979 (23.8%)	28 (22.8%)	2 (28.6%)	
7–8	11,906 (49.0%)	3049 (48.5%)	6851 (49.7%)	1965 (47.8%)	40 (32.5%)	1 (14.3%)	
9–10	7267 (29.9%)	2425 (38.5%)	4036 (29.3%)	792 (19.3%)	13 (10.6%)	1 (14.3%)	
Age (years)	55.2 ± 17.1	44.1 ± 15.1	58.4 ± 16.1	61.1 ± 15.7	63.5 ± 16.1	55.7 ± 18.5	<0.001
Women	13,020 (53.5%)	4121 (65.5%)	7232 (52.5%)	1626 (39.5%)	40 (32.5%)	1 (14.3%)	<0.001
BMI (kg/m^2^)	22.4 ± 3.8	21.8 ± 3.0	22.8 ± 3.8	22.3 ± 4.3	21.9 ± 4.1	22.2 ± 3.0	<0.001
Estimated blood loss							
Median [IQR] (ml)	40 [10–190]	26 [7–100]	40 [10–200]	66 [17–275]	180 [30–1035]	381 [28–1750]	<0.001
>1000	1337 (5.5%)	198 (3.1%)	781 (5.7%)	325 (7.9%)	31 (25.2%)	2 (28.6%)	<0.001
601–1000	1317 (5.4%)	283 (4.5%)	775 (5.6%)	249 (6.1%)	9 (7.3%)	1 (14.3%)	
101–600	5324 (21.9%)	1067 (17.0%)	3067 (22.3%)	1156 (28.1%)	32 (26.0%)	2 (28.6%)	
<100	16,340 (67.2%)	4745 (75.4%)	9159 (66.5%)	2383 (57.9%)	51 (41.5%)	2 (28.6%)	
Lowest mean arterial pressure							
Mean ± SD (mmHg)	56.9 ± 12.9	58.6 ± 11.9	56.8 ± 12.9	54.9 ± 13.7	53.7 ± 13.9	46.7 ± 22.0	<0.001
<40	1247 (5.1%)	211 (3.4%)	672 (4.9%)	345 (8.4%)	16 (13.0%)	3 (42.9%)	<0.001
40–54	9956 (40.9%)	2190 (34.8%)	5801 (42.1%)	1903 (46.3%)	60 (48.8%)	2 (28.6%)	
55–69	9767 (40.2%)	2880 (45.8%)	5479 (39.8%)	1373 (33.4%)	35 (28.5%)	–	
>70	3348 (13.8%)	1012 (16.1%)	1830 (13.3%)	492 (12.0%)	12 (9.8%)	2 (28.6%)	
Lowest heart rate							
Mean ± SD (bpm)	54.6 ± 10.8	53.0 ± 9.8	54.3 ± 10.2	57.7 ± 12.9	67.1 ± 19.2	71.9 ± 12.0	<0.001
>85	426 (1.8%)	77 (1.2%)	182 (1.3%)	143 (3.5%)	23 (18.7%)	1 (14.3%)	<0.001
76–85	707 (2.9%)	117 (1.9%)	359 (2.6%)	214 (5.2%)	15 (12.2%)	2 (28.6%)	
66–75	2189 (9.0%)	433 (6.9%)	1181 (8.6%)	550 (13.4%)	23 (18.7%)	2 (28.6%)	
56–65	5951 (24.5%)	1330 (21.1%)	3444 (25.0%)	1157 (28.1%)	19 (15.4%)	1 (14.3%)	
<55	15,045 (61.9%)	4336 (68.9%)	8616 (62.5%)	2049 (49.8%)	43 (35.0%)	1 (14.3%)	
Emergency procedure	2525 (10.4%)	643 (10.2%)	1250 (9.1%)	567 (13.8%)	59 (48.0%)	6 (85.7%)	<0.001
Operative duration (h)	2.7 ± 1.9	2.4 ± 1.8	2.8 ± 1.9	3.0 ± 1.9	2.6 ± 1.9	2.0 ± 1.1	<0.001
Anesthesia duration (h)	3.7 ± 2.2	3.3 ± 2.1	3.8 ± 2.2	4.2 ± 2.2	3.5 ± 2.2	2.9 ± 1.5	<0.001
30-Day mortality	123 (0.51%)	2 (0.03%)	40 (0.29%)	63 (1.53%)	16 (13.01%)	2 (28.57%)	<0.001

**Table 4:** The sAs combined with ASA-PS (SASA) according to perioperative factors and 30-day mortality

	Total (*N* = 24,318)	0–8 (*N* = 119)	9–12 (*N* = 2002)	13–16 (*N* = 12,687)	17–20 (*N* = 9510)	*P* value
ASA-PS						
1	6293 (25.9%)	–	13 (0.6%)	806 (6.4%)	5474 (57.6%)	<0.001
2	13,782 (56.7%)	2 (1.7%)	633 (31.6%)	9111 (71.8%)	4036 (42.4%)	
3	4113 (16.9%)	70 (58.8%)	1286 (64.2%)	2757 (21.7%)	–	
4	123 (0.5%)	42 (35.3%)	68 (3.4%)	13 (0.1%)	–	
5	7 (0.0%)	5 (4.2%)	2 (0.1%)	–	–	
SAS						
0–2	156 (0.6%)	91 (76.5%)	65 (3.2%)	–	–	<0.001
3–4	1086 (4.5%)	26 (21.8%)	888 (44.4%)	172 (1.4%)	–	
5–6	3903 (16.0%)	2 (1.7%)	1007 (50.3%)	2894 (22.8%)	–	
7–8	11,906 (49.0%)	–	41 (2.0%)	8816 (69.5%)	3049 (32.1%)	
9–10	7267 (29.9%)	–	1 (0.0%)	805 (6.3%)	6461 (67.9%)	
Age (years)	55.2 ± 17.1	56.6 ± 16.8	58.6 ± 16.7	58.4 ± 16.4	50.2 ± 17.0	<0.001
Women	13,020 (53.5%)	39 (32.8%)	903 (45.1%)	6468 (51.0%)	5610 (59.0%)	<0.001
BMI (kg/m^2^)	22.4 ± 3.8	22.1 ± 4.0	22.6 ± 4.3	22.6 ± 3.9	22.1 ± 3.4	<0.001
Estimated blood loss						
Median [IQR] (ml)	40 [10–190]	1640 [940–3225]	612 [132–1195]	60 [15–281]	19 [5–50]	<0.001
>1000	1337 (5.5%)	87 (73.1%)	678 (33.9%)	570 (4.5%)	2 (0.0%)	<0.001
601–1000	1317 (5.4%)	13 (10.9%)	331 (16.5%)	908 (7.2%)	65 (0.7%)	
101–600	5324 (21.9%)	14 (11.8%)	568 (28.4%)	3795 (29.9%)	947 (10.0%)	
≤100	16,340 (67.2%)	5 (4.2%)	425 (21.2%)	7414 (58.4%)	8496 (89.3%)	
Lowest mean arterial pressure						
Mean ± SD (mmHg)	56.9 ± 12.9	42.7 ± 11.4	48.6 ± 11.3	54.2 ± 12.1	62.4 ± 11.9	<0.001
<40	1247 (5.1%)	44 (37.0%)	407 (20.3%)	683 (5.4%)	113 (1.2%)	<0.001
40–54	9956 (40.9%)	67 (56.3%)	1143 (57.1%)	6905 (54.4%)	1841 (19.4%)	
55–69	9767 (40.2%)	6 (5.0%)	381 (19.0%)	4030 (31.8%)	5350 (56.3%)	
≥70	3348 (13.8%)	2 (1.7%)	71 (3.5%)	1069 (8.4%)	2206 (23.2%)	
Lowest heart rate						
Mean ± SD (bpm)	54.6 ± 10.8	85.9 ± 15.7	67.6 ± 14.5	55.6 ± 10.2	50.2 ± 6.8	<0.001
>85	426 (1.8%)	58 (48.7%)	237 (11.8%)	131 (1.0%)		<0.001
76–85	707 (2.9%)	36 (30.3%)	285 (14.2%)	365 (2.9%)	21 (0.2%)	
66–75	2189 (9.0%)	18 (15.1%)	568 (28.4%)	1433 (11.3%)	170 (1.8%)	
56–65	5951 (24.5%)	5 (4.2%)	563 (28.1%)	3959 (31.2%)	1424 (15.0%)	
≤55	15,045 (61.9%)	2 (1.7%)	349 (17.4%)	6799 (53.6%)	7895 (83.0%)	
Emergency procedure	2525 (10.4%)	95 (79.8%)	545 (27.2%)	1342 (10.6%)	543 (5.7%)	<0.001
Operative duration (h)	2.7 ± 1.9	3.3 ± 2.4	3.6 ± 2.4	2.9 ± 1.9	2.3 ± 1.5	<0.001
Anesthesia duration (h)	3.7 ± 2.2	4.2 ± 2.8	4.7 ± 2.9	4.0 ± 2.2	3.2 ± 1.8	<0.001
30-Day mortality	123 (0.51%)	21 (17.65%)	57 (2.84%)	42 (0.33%)	3 (0.03%)	<0.001

### 30-Day mortality of the sAs, ASA-PS, and SASA

As the sAs, ASA-PS, and SASA indicated more severe conditions, mortality tended to be significantly higher (*P* < 0.001) (Tables 2, 3, 4). In addition, the risk of death was elevated by 3.65 for every 2-point decrease in the sAs, by 6.4 for every 1-point increase in the ASA-PS score, and by 9.56 for every 4-point decrease in the SASA (*P* < 0.001) (Table 5).

**Table 5:** Multivariate logistic regression model for 30-day mortality

	Univariate analysis		Multivariate analysis	
	Unadjusted OR (95% CI)	*P* value	Adjusted OR (95% CI)	*P* value
sAs				
Per 2 score decrease	3.65 (3.10–4.41)	<0.001	2.72 (2.28–3.24)	<0.001
ASA-PS				
Per 1 score increase	6.40 (4.95–8.30)	<0.001	3.99 (3.05–5.21)	<0.001
SASA				
Per 4 score decrease	9.56 (7.52–12.16)	<0.001		

*CI* confidence interval

### ROC curve of the SASA

**Fig. 1:**
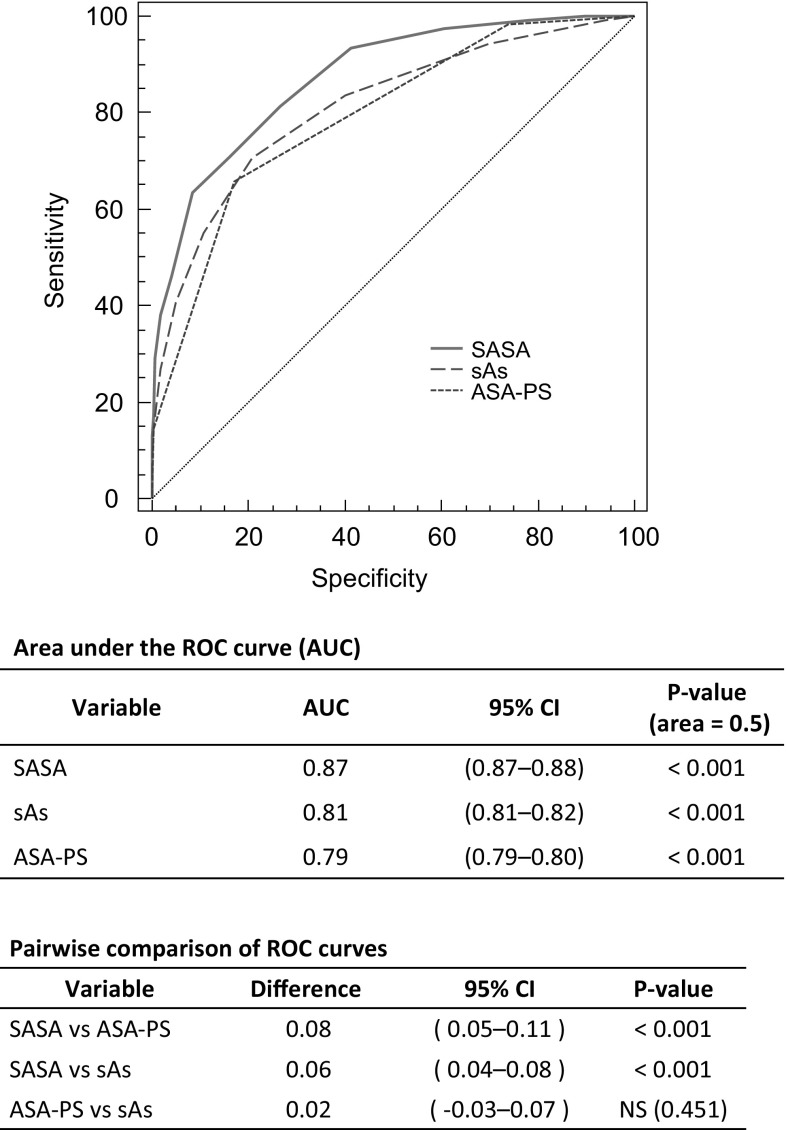
ROC curves of three scores of 30-day mortality

The ROC curves of the sAs and ASA-PS alone also individually demonstrated that the sAs and ASA-PS were highly valid [area under the curve (AUC) = 0.81 for sAs and 0.79 for ASA-PS, *P* < 0.001]. There was no difference between these 2 scoring systems (*P* = 0.451). However, the SASA, which combined them, was even more valid (AUC = 0.87, *P* < 0.001) (Fig. 1).

## Discussion

Both sAs and ASA-PS were found to be very useful scoring systems for predicting postoperative 30-day mortality, but the SASA demonstrated a predictive ability that was superior to those scoring systems. Our study differs from previous reports on the sAs in several aspects. First, the proportion of patients with an ASA-PS score of 3 or higher is small [9, 10]. Second, patients undergoing cardiovascular surgery were excluded. Finally, the National Surgical Quality Improvement Program (NSQIP) [11], on which the sAs calculation is based, excludes patients undergoing endoscopic surgery, which was included in our study. These aspects might have contributed to the postoperative 30-day mortality being lower in our study than in previous reports [9, 10]. However, the sAs still demonstrated a high predictive ability in our study, and its wide versatility that is not affected by differences in patient characteristics and target facilities was consistent with previous reports [12]. Meanwhile, the ASA-PS, which was not originally developed as a risk indicator, has been reported to be useful for predicting outcomes [13–16]. In our study, the ASA-PS also demonstrated a high predictive ability that was comparable with that described in previous reports. Although this scoring system has the limitation of reflecting only preoperative patient status [17], our results can help anesthesiologists to realize that the ASA-PS, which we use usually, is highly useful for predicting postoperative patient status.

In this study, we developed a new scoring system to evaluate both preoperative and intraoperative patient statuses. The SASA, which combines the sAs and ASA-PS, demonstrated a much higher predictive ability, compared with either the sAs or ASA-PS. The accuracy of the sAs and ASA-PS is reportedly improved by addition of factors such as age, surgical invasiveness, and respiratory complications [18–22]. Although such modifications do not make the calculation of the scores as complex as that of the APACHE and POSSUM, the calculation of those still remains complex. The modified scoring systems have not been widely adopted. The SASA is a scoring system that is easy to calculate and combines the sAs and ASA-PS, each of which is highly useful. While the calculation of the SASA is simple, its predictive ability appears to be comparable with the previously reported predictive ability of the POSSUM and APACHE [23]. In the SASA, the ASA-PS reflects preoperative patient status, and the sAs reflects intraoperative patient status. Thus, the SASA is expected to clearly and comprehensively indicate perioperative risk in patients. Moreover, because the ASA-PS, which is criticized for its predominant influence of subjective elements, is complemented by the addition of the sAs, which is calculated only with objective elements, the SASA scoring system is extremely practical. For example, in cases of cesarean delivery, in which hypotension is prevalent, if the volume of blood loss includes amniotic fluid, the severity based on the sAs alone will be extremely high. However, addition of the ASA-PS reduces such false-positive results.

This study has some limitations. First, it is based on data collected at a single large academic center. Because data compiled by multicenter registries, such as NSQIP and the National Anesthesia Clinical Outcomes Registry (NACOR) [24], are not used, further studies are needed to determine whether our proposed SASA will also be as useful at other facilities, as shown by the results of this study. Although the SASA originates from Japan, we hope that wide international validation will follow; the sAs originally came from a single academic center, and its versatility has been widely reported since then. Moreover, further studies may also be needed on the usefulness of the SASA in patients undergoing cardiovascular surgery and those aged 16 years or younger, who were excluded from this study. In our study the number of patients classified as ASA-PS IV and V was so small that further studies may be needed in a patient population which has an equal distribution of ASA-PS, to show the versatility of SASA.

In summary, the sAs and ASA-PS were shown to be extremely useful for predicting mortality within 30 days of surgery. An even higher predictive ability was demonstrated by the SASA, which combines these simple and effective scoring systems. We expect that the SASA will be widely used as a new easy scoring system for predicting prognosis, allowing a comprehensive assessment of perioperative patient status and automatic calculation of scores at the end of entering data into electronic anesthesia charts.
